# Draft genome sequence of *Bosea* sp. WAO an arsenite and sulfide oxidizer isolated from a pyrite rock outcrop in New Jersey

**DOI:** 10.1186/s40793-018-0312-4

**Published:** 2018-04-10

**Authors:** Alexandra B. Walczak, Nathan Yee, Lily Y. Young

**Affiliations:** 1 Division of Life Sciences, Rutgers University, The State University of New Jersey, Piscataway, New Jersey USA; 2 Department of Environmental Sciences, Rutgers University, The State University of New Jersey, New Brunswick, New Jersey USA

**Keywords:** Neutrophilic sulfur oxidizer, *Sox*, Arsenite oxidase gene, *Aio*, Geomicrobiology, Microbe-mineral interactions, carbon fixation RuBisCO

## Abstract

This genome report describes the draft genome and physiological characteristics of *Bosea* sp. WAO (=DSM 102914), a novel strain of the genus *Bosea* in the family *Bradyrhizobiaceae*. *Bosea* sp. WAO was isolated from pulverized pyritic shale containing elevated levels of arsenic. This aerobic, gram negative microorganism is capable of facultative chemolithoautotrophic growth under aerobic conditions by oxidizing the electron donors arsenite, elemental sulfur, thiosulfate, polysulfide, and amorphous sulfur. The draft genome is of a single circular chromosome 6,125,776 bp long consisting of 21 scaffolds with a G + C content of 66.84%. A total 5727 genes were predicted of which 5665 or 98.92% are protein-coding genes and 62 RNA genes. We identified the genes *aioA* and *aioB*, which encode the large and small subunits of the arsenic oxidase respectively. We also identified the genes for the complete sulfur oxidation pathway *sox* which is used to oxidize thiosulfate to sulfate.

## Introduction

*Bosea* sp. WAO (white arsenic oxidizer) was enriched from a pulverized sample of weathered black shale obtained from an outcropping near Trenton, NJ that contained high levels of arsenic [1]. *Bosea* sp. WAO belongs to the class *Alphaproteobacteria* and family *Bradyrhizobiaceae* which currently consists of 12 genera: *Bradyrhizobium*, *Afipia*, *Agromonas*, *Balneimonas*, *Blastobacter*, *Bosea*, *Nitrobacter*, *Oligotropha*, *Rhodoblastus*, *Rhodopseudomomonas*, *Salinarimonas*, and *Tardiphaga* [2]. This phenotypically diverse family is composed of microorganisms that are involved in nitrogen cycling, human diseases, phototropism in non-sulfur environments, plant commensalism, and chemolithoautotrophic growth [2]. 16S rRNA gene analysis of the *Bradyrhizobiaceae* family indicates that the *Bosea* genus is most closely related to the genus *Salinarimonas* which currently consists of two species, *Salinarimonas rosea* and *Salinarmonas ramus* [2]. The microorganisms belonging to the genus *Bosea* have been isolated from a variety of environments such as soils, sediments, hospital water systems, and digester sludge [3–5]. The type strain *Bosea thiooxidans* BI-42^T^is capable of thiosulfate oxidation and the initial genus definition included this characteristic [3]. In 2003 La Scola emended the genus description to remove thiosulfate oxidation as a key descriptor after isolation of several other *Bosea* spp. that were unable to oxidized thiosulfate [4]. These organisms have a very diverse metabolism but their common characteristics include being Gram-negative, aerobic, rod shaped, motile, good growth between 25 to 35 °C, intolerant to salt concentrations above 6% NaCl and have been described to be heterotrophic [3–5]. Using selective enrichment and isolation techniques with arsenite [As(III)] as the sole electron donor *Bosea* sp. WAO was isolated under autotrophic conditions [1]. Here we summarize the physiological features together with the draft genome sequence and data analysis of *Bosea* sp. WAO.

## Organism information

### Classification and features

**Fig. 1:**
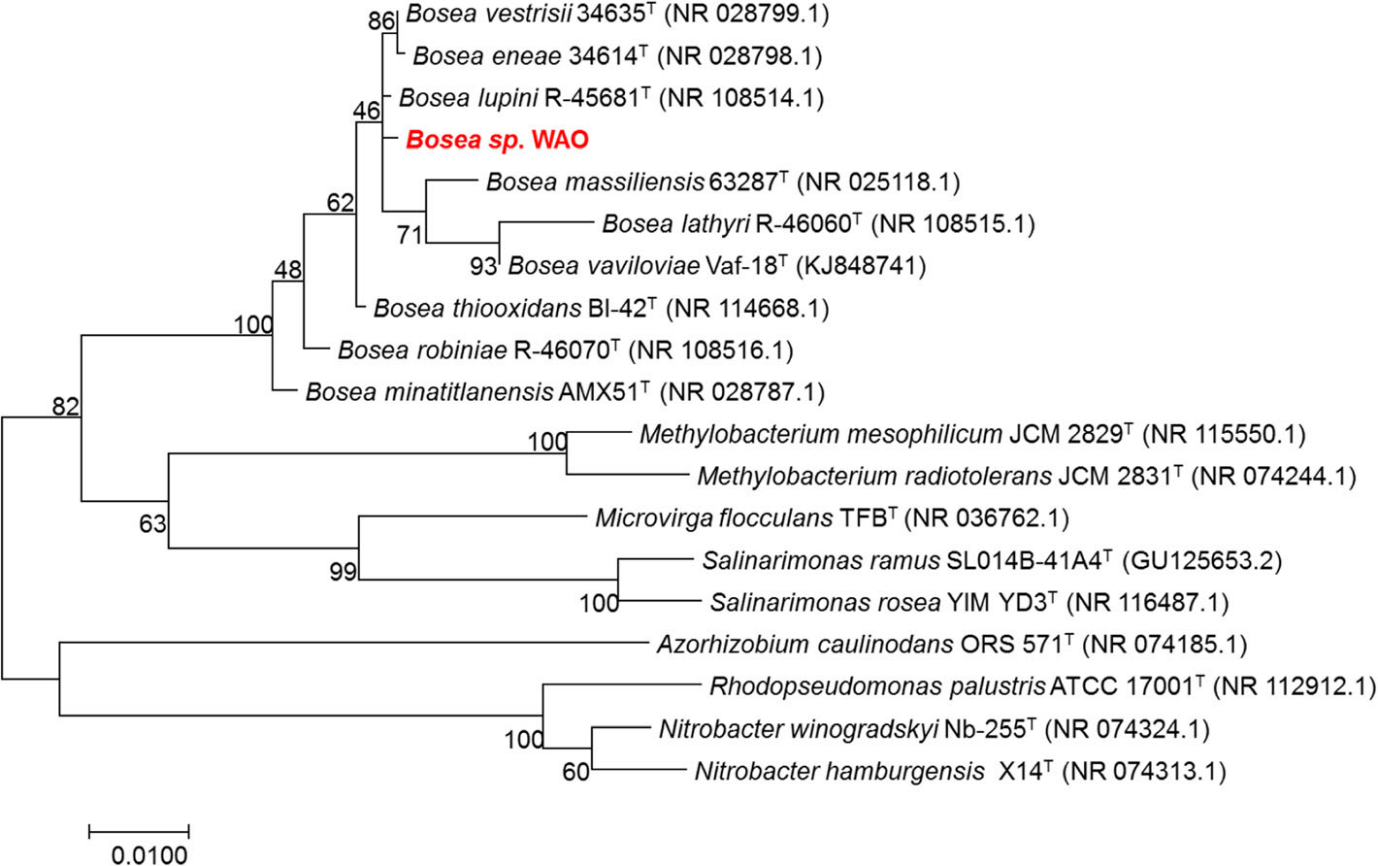
Molecular Phylogenetic analysis by Maximum Likelihood method of the 16S rRNA gene. A Phylogenetic tree highlighting the position of *Bosea* sp. WAO relative to the other *Bosea* spp. based on the 16 s rRNA gene. The evolutionary history was inferred by using the Maximum Likelihood method based on the Tamura-Nei model [19]. The tree with the highest log likelihood (− 4792.5378) is shown. The percentage of trees in which the associated taxa clustered together is shown next to the branches. Initial tree(s) for the heuristic search were obtained automatically by applying Neighbor-Join and BioNJ algorithms to a matrix of pairwise distances estimated using the Maximum Composite Likelihood (MCL) approach, and then selecting the topology with superior log likelihood value. The tree is drawn to scale, with branch lengths measured in the number of substitutions per site. The analysis involved 19 nucleotide sequences. All positions containing gaps and missing data were eliminated. There were a total of 1376 positions in the final dataset. Evolutionary analyses were conducted in MEGA7 [20]. Type strains are indicated with a superscript T

**Fig. 2:**
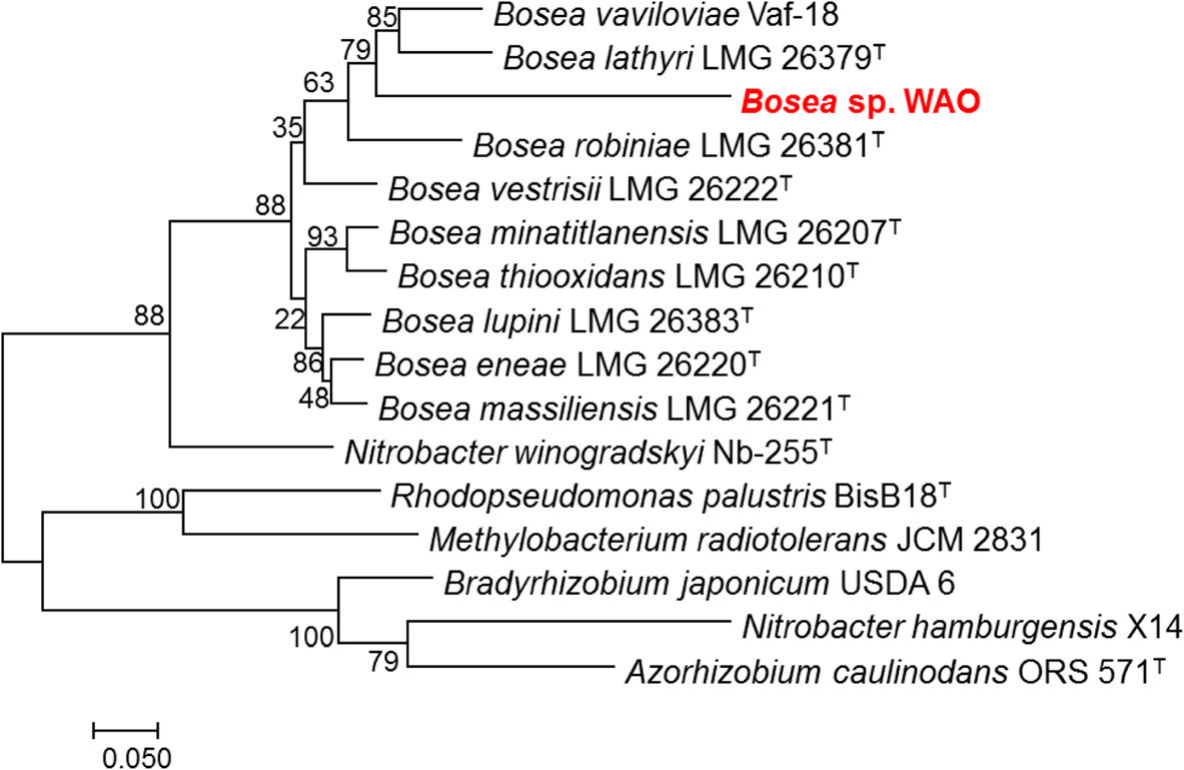
Molecular Phylogenetic analysis by Maximum Likelihood method of aligned concatenated *atpD*, *dnaK*, *gyrB*, *recA*, and *rpoB*. The evolutionary history was inferred by using the Maximum Likelihood method based on the Tamura-Nei model [1]. The tree with the highest log likelihood (− 13842.8588) is shown. The percentage of trees in which the associated taxa clustered together is shown next to the branches. Initial tree(s) for the heuristic search were obtained automatically by applying Neighbor-Join and BioNJ algorithms to a matrix of pairwise distances estimated using the Maximum Composite Likelihood (MCL) approach, and then selecting the topology with superior log likelihood value. The tree is drawn to scale, with branch lengths measured in the number of substitutions per site. The analysis involved 16 nucleotide sequences. All positions containing gaps and missing data were eliminated. There were a total of 1413 positions in the final dataset. Evolutionary analyses were conducted in MEGA7 [21]

**Fig. 3:**
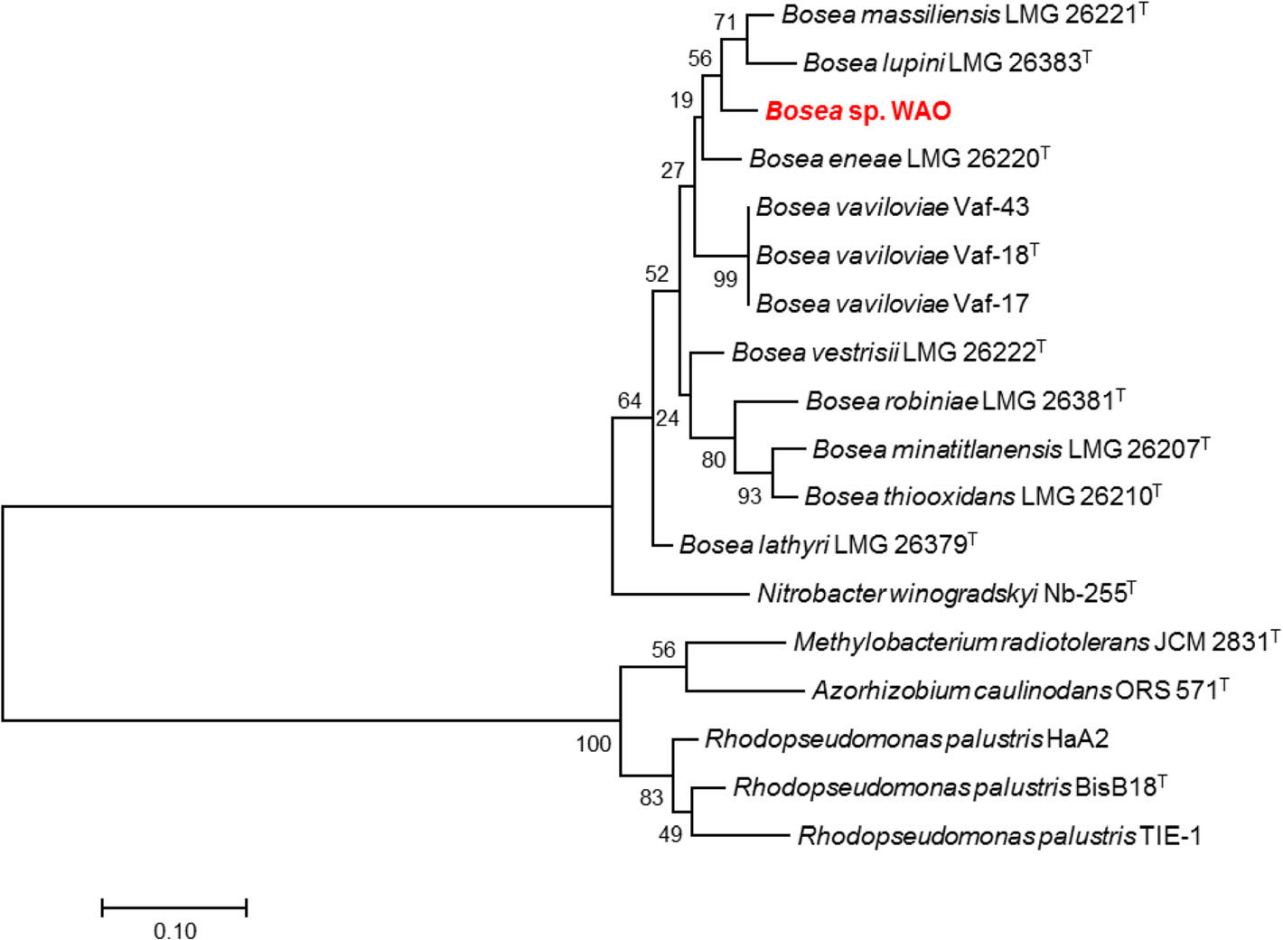
Molecular Phylogenetic analysis by Maximum Likelihood method of the *aptD* gene. The evolutionary history was inferred by using the Maximum Likelihood method based on the Tamura-Nei model [19]. A Phylogenetic tree highlighting the position of *Bosea* sp. WAO relative to the other *Bosea* spp. and related organisms based on the *aptD* gene. The tree with the highest log likelihood (− 2412.0185) is shown. The percentage of trees in which the associated taxa clustered together is shown next to the branches. Initial tree(s) for the heuristic search were obtained automatically by applying Neighbor-Join and BioNJ algorithms to a matrix of pairwise distances estimated using the Maximum Composite Likelihood (MCL) approach, and then selecting the topology with superior log likelihood value. The tree is drawn to scale, with branch lengths measured in the number of substitutions per site. The analysis involved 18 nucleotide sequences. All positions containing gaps and missing data were eliminated. There were a total of 361 positions in the final dataset. Evolutionary analyses were conducted in MEGA7 [20]. Type strains are indicated with a superscript T

**Fig. 4:**
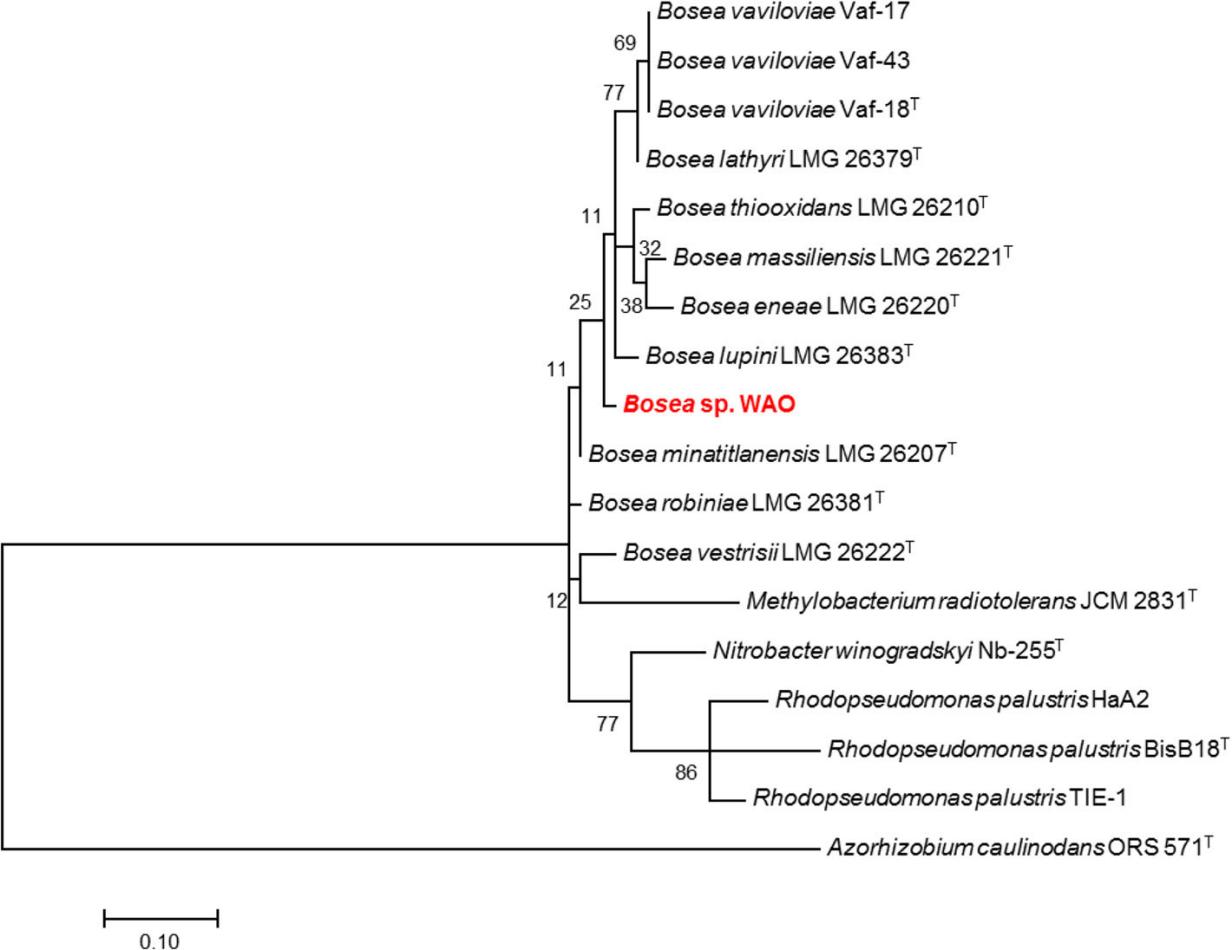
Molecular Phylogenetic analysis by Maximum Likelihood method of the *dnaK* gene. The evolutionary history was inferred by using the Maximum Likelihood method based on the Tamura-Nei model [19]. The tree with the highest log likelihood (− 613.9292) is shown. The percentage of trees in which the associated taxa clustered together is shown next to the branches. Initial tree(s) for the heuristic search were obtained automatically by applying Neighbor-Join and BioNJ algorithms to a matrix of pairwise distances estimated using the Maximum Composite Likelihood (MCL) approach, and then selecting the topology with superior log likelihood value. The tree is drawn to scale, with branch lengths measured in the number of substitutions per site. The analysis involved 18 nucleotide sequences. Codon positions included were 1st + 2nd + 3rd + Noncoding. All positions containing gaps and missing data were eliminated. There were a total of 103 positions in the final dataset. Evolutionary analyses were conducted in MEGA7 [20]. Type strains are indicated with a superscript T

**Fig. 5:**
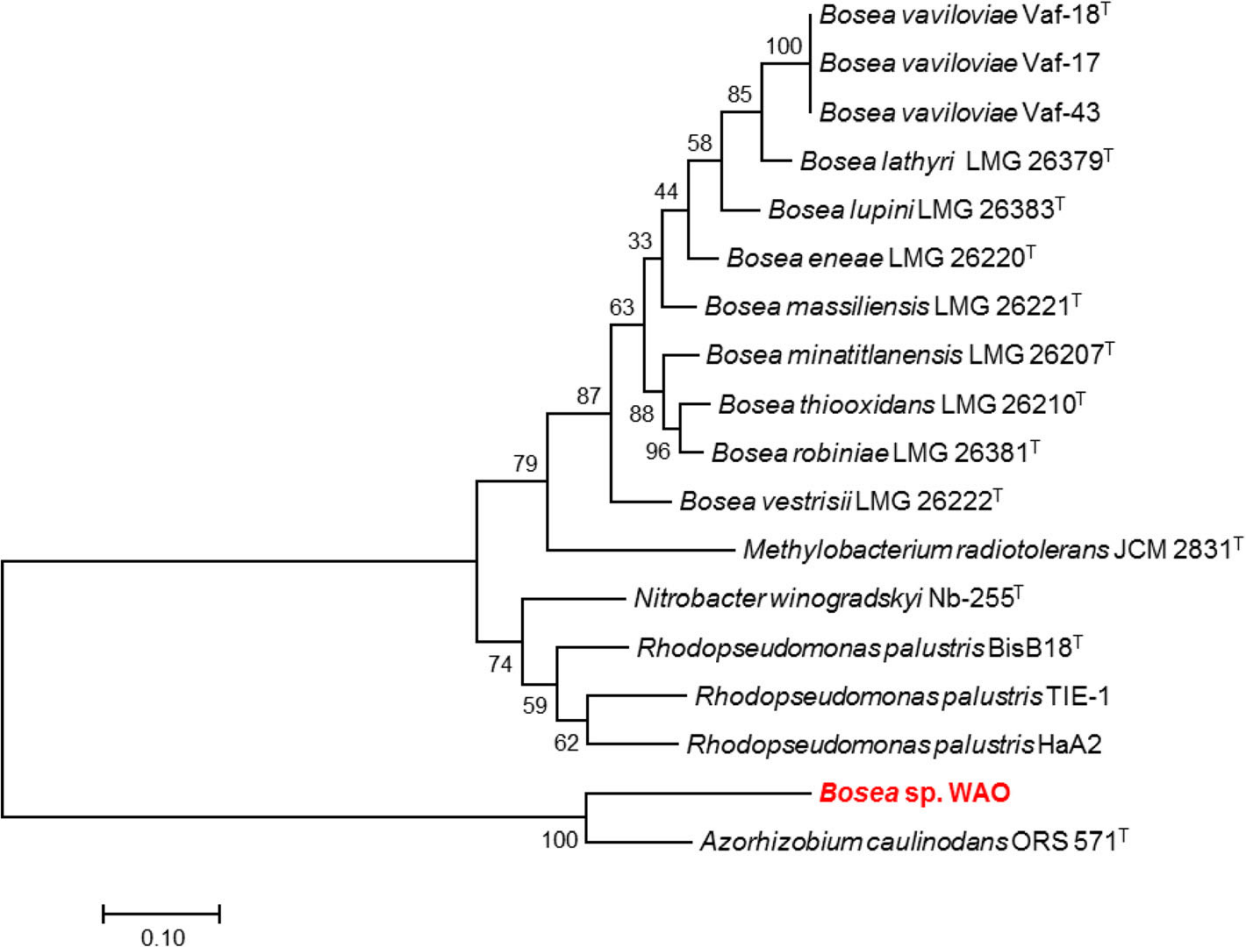
Molecular Phylogenetic analysis by Maximum Likelihood method of gyrB gene. The evolutionary history was inferred by using the Maximum Likelihood method based on the Tamura-Nei model [19]. The tree with the highest log likelihood (− 4279.1901) is shown. The percentage of trees in which the associated taxa clustered together is shown next to the branches. Initial tree(s) for the heuristic search were obtained automatically by applying Neighbor-Join and BioNJ algorithms to a matrix of pairwise distances estimated using the Maximum Composite Likelihood (MCL) approach, and then selecting the topology with superior log likelihood value. The tree is drawn to scale, with branch lengths measured in the number of substitutions per site. The analysis involved 18 nucleotide sequences. Codon positions included were 1st + 2nd + 3rd + Noncoding. All positions containing gaps and missing data were eliminated. There were a total of 508 positions in the final dataset. Evolutionary analyses were conducted in MEGA7 [20]. Type strains are indicated with a superscript T

**Fig. 6:**
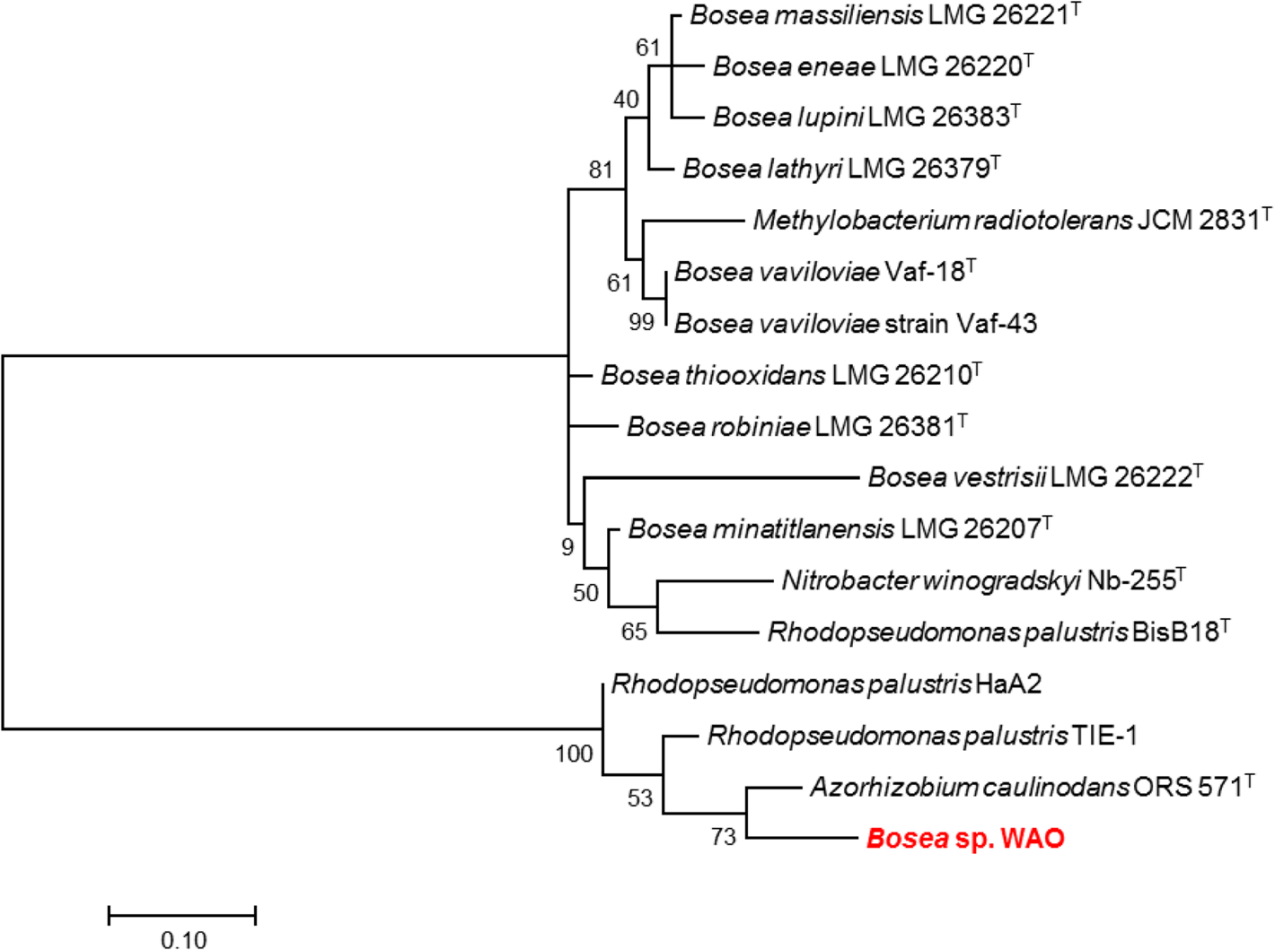
Molecular Phylogenetic analysis by Maximum Likelihood method of *recA* gene. The evolutionary history was inferred by using the Maximum Likelihood method based on the Tamura-Nei model [19]. The tree with the highest log likelihood (− 1263.1252) is shown. The percentage of trees in which the associated taxa clustered together is shown next to the branches. Initial tree(s) for the heuristic search were obtained automatically by applying Neighbor-Join and BioNJ algorithms to a matrix of pairwise distances estimated using the Maximum Composite Likelihood (MCL) approach, and then selecting the topology with superior log likelihood value. The tree is drawn to scale, with branch lengths measured in the number of substitutions per site. The analysis involved 17 nucleotide sequences. Codon positions included were 1st + 2nd + 3rd + Noncoding. All positions containing gaps and missing data were eliminated. There were a total of 190 positions in the final dataset. Evolutionary analyses were conducted in MEGA7 [20]. Type strains are indicated with a superscript T

**Fig. 7:**
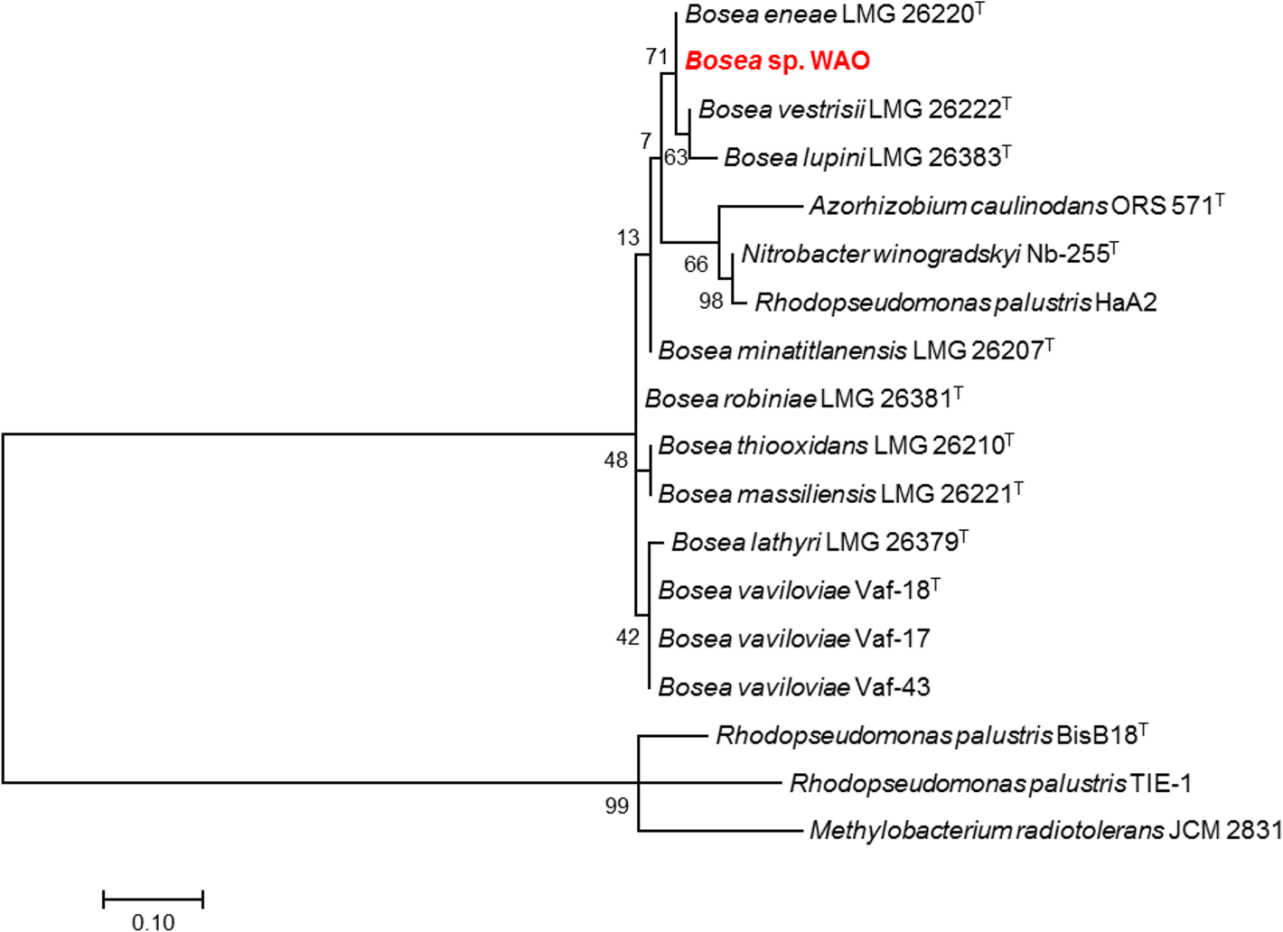
Molecular Phylogenetic analysis by Maximum Likelihood method of *rpoB* gene. The evolutionary history was inferred by using the Maximum Likelihood method based on the Tamura-Nei model [19]. The tree with the highest log likelihood (− 419.8311) is shown. The percentage of trees in which the associated taxa clustered together is shown next to the branches. Initial tree(s) for the heuristic search were obtained automatically by applying Neighbor-Join and BioNJ algorithms to a matrix of pairwise distances estimated using the Maximum Composite Likelihood (MCL) approach, and then selecting the topology with superior log likelihood value. The tree is drawn to scale, with branch lengths measured in the number of substitutions per site. The analysis involved 18 nucleotide sequences. Codon positions included were 1st + 2nd + 3rd + Noncoding. All positions containing gaps and missing data were eliminated. There were a total of 76 positions in the final dataset. Evolutionary analyses were conducted in MEGA7 [20]. Type strains are indicated with a superscript T

The genus *Bosea* has nine species with validly published names isolated from various environments: *B. thiooxidans* BI-42^T^ (AF508803) from agricultural soil [3], *B. eneae* 34614^T^ (AF288300), *B. vestrisii* 34635^T^ (AF288306), and *B. massiliensis* 63287^T^ (AF288309) from a hospital water system [4], *B. minatitlanensis* AMX51^T^ (AF273081) from anaerobic digester sludge [5] *B. lupini* R-45681^T^ (FR774992), *B. lathyri* R-46060^T^ (FR774993), and *B. robiniae* R-46070^T^ (FR774994) from the root nodules of legumes [6], and *B. vaviloviae* Vaf-18^T^(KJ848741) from the root nodules of *Vavilovia formosa* [7]. Strain WAO’s previously published identity was confirmed using the EzTaxon server [8]. The highest 16S rRNA pairwise similarities for strain WAO were found with the type strains *B. vestrisii* 34635^T^ (99.72%), *B. eneae* 34614^T^ (99.65%), *B. lupini* R-45681^T^ (99.65%), *B. thiooxidans* BI-42^T^ (99.24%), *B. robiniae* R-46070^T^ (98.88%), *B. massiliensis* 63287^T^ (98.81%), *B. minatitlanensis* AMX51^T^ (98.48%) and *B. lathyri* R-46060^T^ (98.18%). Phylogenetic analysis based on the 16S rRNA gene of *Bosea* spp. and phylogenetically related organisms placed *Bosea* sp. WAO closest to the type strain *B. lupini* DSM 26673^T^ with *B. vestrisii* 34635^T^ and *B. eneae* 34614^T^ in the same cluster (Fig. 1, Table 1). An average nucleotide identity analysis (ANI) score between strain WAO and *B. lupini* DSM 26673^T^ of 84.64% was computed using IMG/ER [9]. This value is lower than the ANI species demarcation threshold range (95–96%) [10]. To further identify *Bosea* sp. WAO to the species level phylogenic trees based on the housekeeping genes *atpD*, *dnaK*, *recA*, *gyrB* and *rpoB* were produced from available *Bosea* and related *Bradyrhizobiaceae* type strains using MEGA7 (Figs. 2, 3, 4, 5, 6 and 7). Strain WAO did not consistently group with any of the type strains for all five genes further suggesting that it is a separate species. The ability of *B. lupini* to oxidize thiosulfate has not been determined [6]; however, *B. vestrisii*, *B. eneae*, and *B. massiliensis* have been determined to not oxidize thiosulfate to sulfate [4]. These results suggest that strain WAO represents a distinct species in the genus *Bosea*.

**Table 1:** Classification and general features of *Bosea* sp. WAO [22]

MIGS ID	Property	Term	Evidence code^a^
	Classification	Domain *Bacteria*	TAS [23, 24]
		Phylum *Proteobacteria*	TAS [25]
		Class *Alphaproteobacteria*	TAS [26, 27]
		Order *Rhizobiales*	TAS [27, 28]
		Family *Bradyrhizobiaceae*	TAS [27, 29]
		Genus *Bosea*	TAS [3, 30]
		Species *Bosea* sp.	TAS [24]
		Strain: WAO (DSM 102914)	TAS [1]
	Gram stain	Negative	IDA
	Cell shape	Rod	TAS [1]
	Motility	Motile	TAS [1]
	Sporulation	Not reported	NAS
	Temperature range	Mesophile	IDA
	Optimum temperature	25–30 °C	IDA
	pH range; Optimum	6–9; 8	IDA
	Carbon source	D-glucose, lactose, acetate, bicarbonate	TAS [1]
MIGS-6	Habitat	Terrestrial, Black shale	TAS [1]
MIGS-6.3	Salinity	No growth with > 3.5% NaCl (*w*/*v*)	IDA
MIGS-22	Oxygen requirement	Aerobic	TAS [1]
MIGS-15	Biotic relationship	free-living	TAS [1]
MIGS-14	Pathogenicity	Not reported	NAS
MIGS-4	Geographic location	Lockatong formation, New Jersey, USA	TAS [1]
MIGS-5	Sample collection	2005	IDA
MIGS-4.1	Latitude	40.289329	IDA
MIGS-4.2	Longitude	− 74.814366	IDA
MIGS-4.4	Altitude	60 m	IDA

These evidence codes are from the Gene Ontology project [31]

^a^Evidence codes

*IDA* Inferred from Direct Assay, *TAS* Traceable Author Statement (i.e., a direct report exists in the literature), *NAS* Non-traceable Author Statement (i.e., not directly observed for the living, isolated sample, but based on a generally accepted property for the species, or anecdotal evidence)

#### Extended feature descriptions

**Fig. 8:**
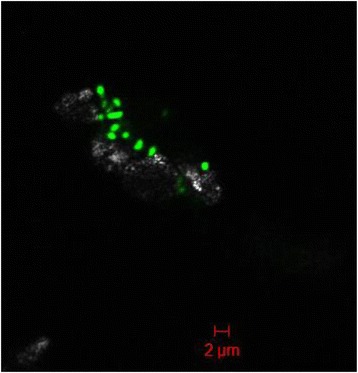
Confocal microscopy of *Bosea* sp. WAO. *Bosea* sp. WAO (green) was stained with DAPI and imaged growing on the surface of a cadmium sulfide particle (faint white/grey) in a mostly black background

*Bosea* sp. WAO cells are Gram-negative, aerobic, motile, and rod shaped. Colonies on trypticase soy agar are smooth, mucoid, round, convex, and beige with a diameter as large as 10 mm after 2 weeks at 30 °C. Colonies on minimal salts medium supplemented with 5 mM sodium thiosulfate are smooth, round, white and only grow to a diameter of 2 mm after 2 weeks at 30 °C. Optimal growth occurs at a temperature range from + 25 to 30 °C and pH 6 to 9 with an optimum at pH 8 (Table 1). Growth did not occur at salinity > 3.5% *w*/*v* of NaCl. Cells will grow freely floating or attached to a mineral surface as shown in Fig. 8.

Strain WAO is a strict aerobe that can grow heterotrophically on acetate, glucose, and lactate in addition to autotrophically on carbon dioxide with the electron donors arsenite, thiosulfate, polysulfide, and elemental sulfur. The organism is also able to grow on the mineral arsenopyrite (FeAsS) by oxidizing both the arsenic and sulfur to produce sulfate and arsenate. No growth was observed under aerobic conditions with the aromatic compounds phenol, benzoate or ferulic acid or with the electron donors sulfite, ammonium, nitrite, selenite, or chromium(III). This organism was enriched from pulverized black shale that contained high levels of arsenic. The initial enrichment cultures using the shale material were amended with 5 mM arsenite and then serially diluted until purity was obtained [1].

## Genome sequencing information

### Genome project history

*Bosea* sp. WAO was selected for sequencing based on the organism’s ability to grow both heterotrophically and chemolithoautotrophically with arsenite and reduced sulfur compounds. Sequencing and assembly was completed at the Rutgers School of Environmental and Biological Sciences Genome Cooperative. A paired-end library was constructed using an Illumina Nextera Kit and sequenced using an Illumina Genome Analyzer IIX (Illumina Inc., San Diego, CA). The sequence assembly was performed using a CLC Genomics Workbench 5.1 (CLC Bio, Cambridge, MA). The draft genome was submitted to NCBI Whole Genome Shotgun (WGS) and to the JGI Integrated Microbial Genomes/ Expert Review (IMG/ER). A summary of the project is shown in Table 2.

**Table 2:** Project information

MIGS ID	Property	Term
MIGS 31	Finishing quality	Draft
MIGS-28	Libraries used	One pair-end
MIGS 29	Sequencing platforms	Illumina Genome Analyzer IIX
MIGS 31.2	Fold coverage	240×
MIGS 30	Assemblers	CLC Genomics Workbench 5.1
MIGS 32	Gene calling method	Glimmer
	Locus Tag	DK26
	Genbank ID	JXTJ00000000
	GenBank Date of Release	January 8, 2016
	GOLD ID	Gp0113237
	BIOPROJECT	PRJNA243637
MIGS 13	Source Material Identifier	DSM 102914
	Project relevance	Environmental, biogeochemical cycling of arsenic and sulfur

### Growth conditions and genomic DNA preparation

A culture of *Bosea* sp. WAO (GeneBank: DQ986321.1, DSM 102914) was grown in a dilute (50% normal strength) trypticase soy broth amended with 5 mM sodium arsenite and 5 mM sodium thiosulfate then incubated at 30 °C on an orbital shaker for maximum oxygen exchange. Once turbid genomic DNA was extracted using the MoBio Powersoil Kit following manufacturer’s directions with the modification that DNA was eluted into 100 uL water instead of buffer.

### Genome sequencing and assembly

A paired-end library was constructed using an Illumina Nextera Kit and sequenced using an Illumina Genome Analyzer IIX (Illumina Inc., San Diego, CA). The sequence assembly was performed using the CLC Genomics Workbench 5.1 (CLC Bio, Cambridge, MA). An average coverage of 240× and a mean read length of 106 bp was obtained. The genome was assembled into 42 contigs with no additional gap closures.

### Genome annotation

Genes were identified using the standard operating procedures of the DOE-JGI Microbial Genome Annotation pipeline [9] and The RAST Server: Rapid Annotation using subsystem technology [11, 12]. JGI-IMG/ER was used to obtain COG identities and overall statistics of the genome. RAST was used to identify functional genes of interest involved in sulfur and arsenic metabolism.

## Genome properties

The draft genome is 6,125,776 bp with 66.84% G + C content. There are 62 RNA genes, 1 each of 5S rRNA, 16S rRNA, and 23S rRNA, and 46 tRNA, plus 13 unclassified RNA (Table 3). Of the predicted 5727 genes, 5665 or 98.92% are protein-coding genes, with 82.77% identified with protein function. The draft genome contains no identified pseudo genes. Of the protein-coding genes 4193 were sorted into COG functional categories. The COG categories are broken down in Table 4. COG analysis assigned a large number of genes to amino acid transport and metabolism (13.76%), transcription (8.13%), inorganic ion transport and metabolism (8.06%), and energy production and conservation (6.97%). *Bosea* sp. WAO has 53 genes encoding for cytochromes alone. RAST subsystem analysis placed 44% of the protein coding genes into subsystem categories with the largest percentage assigned to amino acids and derivatives. The genome sequence was deposited in GenBank ID JXTJ00000000.

**Table 3:** Genome statistics

Attribute	Value	% of total
Genome size (bp)	6,125,776	100.00
DNA coding (bp)	5,469,601	89.29
DNA G + C (bp)	4,094,621	66.84
DNA scaffolds	42	100.00
Total genes	5727	100.00
Protein coding genes	5665	98.92
RNA genes	62	1.08
Pseudo genes	0	0
Genes in internal clusters		
Genes with function prediction	4740	82.77
Genes assigned to COGs	4193	73.21
Genes with Pfam domains	4837	84.46
Genes with signal peptides	635	11.09
Genes with transmembrane helices	1391	24.29
CRISPR repeats	0	0

**Table 4:** Number of genes associated with general COG functional categories

Code	Value	%age	Description
J	212	4.44	Translation, ribosomal structure and biogenesis
A	0	0	RNA processing and modification
K	388	8.13	Transcription
L	112	2.35	Replication, recombination and repair
B	3	0.06	Chromatin structure and dynamics
D	29	0.61	Cell cycle control, cell division, chromosome partitioning
V	109	2.28	Defense mechanisms
T	213	4.46	Signal transduction mechanisms
M	239	5.01	Cell wall/membrane biogenesis
N	75	1.57	Cell motility
U	60	1.26	Intracellular trafficking and secretion
O	175	3.66	Posttranslational modification, protein turnover, chaperones
C	333	6.97	Energy production and conversion
G	276	5.78	Carbohydrate transport and metabolism
E	657	13.76	Amino acid transport and metabolism
F	102	2.14	Nucleotide transport and metabolism
H	233	4.88	Coenzyme transport and metabolism
I	249	5.21	Lipid transport and metabolism
P	385	8.06	Inorganic ion transport and metabolism
Q	155	3.25	Secondary metabolites biosynthesis, transport and catabolism
R	470	9.84	General function prediction only
S	268	5.61	Function unknown
–	1534	26.79	Not in COGs

The total is based on the total number of protein coding genes in the genome

### Extended insights

Ten other genome sequences of *Bosea* spp. are publicly available of which four are validly named and characterized to the species level: *B. thiooxidans* CGMCC 9174 V5_1, *B. lathyri* DSM 26656^T^, *B. lupini* DSM 26673^T^, *B. vaviloviae* strain SD260 and six uncharacterized: *Bosea* sp. 117, *Bosea* sp. UNC402CLCol, *Bosea* sp. LC85, *Bosea* sp. OK403, *Bosea* sp. AAP35, and *Bosea* sp. AAP25. Only *B. thiooxidans* CGMCC 9174 V5_1 and *B. vaviloviae* strain SD260 are complete genomes. Table 5 details the basic characteristics of the ten genomes. The genomes range in size from 4.4 Mb to 6.6 Mb and G + C content between 64 to 68%, a predicated gene number range from 3984 to 6267. *Bosea* *sp*. WAO’s genome size (6.1 Mb), number of predicted genes (5727), number genes with function (4570), and number placed in COGs (4193) are all higher than the average for the draft genomes. However, both the percentage values for genes with functional predication (79.8%) and percentage in COGs (73.2%) are similar to the average values for the draft genomes. *B. thiooxidans* CGMCC 9174 V5_1, *B. vaviloviae* strain SD260, *Bosea* sp. 117 and *Bosea* sp. UNC402CLCol contain pseudo genes. None of the IMG database genomes have been finished with scaffold numbers ranging between 16 and 72.

**Table 5:** Comparison of basic genome features of *Bosea spp*.

Genome Name	Status^a^	Genome Size (Mbp)	G + C Content (%)	Gene Count	No. of protein coding genes w/ function prediction	Percentage (%)	No. of protein coding genes in COGs	Percentage (%)	IMG Taxon ID
*Bosea* sp. WAO [1]	D	6.12	67	5727	4570	79.8	4193	73.2	2615840542
*Bosea* sp. LC85 [32]	PD	6.56	65	6267	4975	79.4	4548	72.6	2609460206
*Bosea sp*. UNC402CLCol	PD	5.61	67	5389	4375	81.1	4067	75.5	2579779168
*Bosea lupini* DSM 26673 [6]	D	6.13	67	5985	4752	79.4	4396	73.4	2634166302
*Bosea sp.* OK403	D	6.64	65	6099	5066	83.1	4396	77.0	2609459641
*Bosea sp*. AAP25	PD	4.14	64	3984	3023	75.9	2651	66.5	2636415410
*Bosea lathyri* DSM 26656 [21]	D	5.91	65	5559	4476	80.5	4120	74.1	2622736433
*Bosea sp*. 117	PD	4.63	68	4344	3639	83.8	3366	77.5	2562617052
*Bosea sp*. AAP35	PD	4.46	66	4298	3435	79.9	3144	73.1	2636415883
*Bosea vaviloviae* strain SD260	F	5.60	66	5487	4260	77.6	3839	69.9	2654587694
*Bosea thiooxidans* CGMCC 9174 V5_1	F	5.46	67	5176	–	–	–	–	N/A

These data were obtained from the IMG/ER platform [9] and NCBI genomes

^a^Status: *D* draft, *PD* permanent draft, *F* finished

#### Arsenite oxidation

**Fig. 9:**
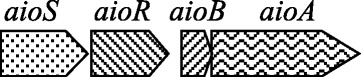
Operon structure for arsenite oxidation viewed 5′-3′ direction on the plus strand. The gene order is the same in both *Bosea* sp. WAO and *Bosea* sp. 117 with a senor histidine kinase, *aioS*, then a transcriptional response regulator, *aioR*, followed by the *aioB* and *aioA* genes

*Bosea* sp. WAO is able to grow under chemolithoautotrophic conditions with arsenite in addition to growing under heterotrophic conditions. Metabolic studies indicated that the organism was able to stoichiometically oxidize the electron donors As(III) to As(V). Aerobic arsenite oxidation occurs using the *aio* genes renamed to reduce confusion from *aso*, *aro* and *aox*, which were formerly used to identify these genes in different organisms [13]. *aioA* encodes for a large molybdopterin containing subunit with a guanosine dinucleotide at the active site and *aioB* encodes for a small Rieske subunit [13–15]. This pathway has a two component regulatory system that includes a sensor histidine kinase encoded by *aioS* (*aoxS*, *aroS*) and a transcriptional regulator encoded by *aioR* (*aoxR*, *aroR*) [13–15]. For the initial publication of *Bosea* sp. WAO, only the large subunit gene for the arsenite oxidation pathway *aioA* (EF015463) was amplified by traditional PCR [1, 16]. Analysis of the genome herein revealed that the arsenite oxidation pathway was complete with *Bosea* sp. WAO possessing both the small subunit *aioB* and reconfirming the large subunit *aioA* in addition to the remaining genes in the pathway. Of the available genomes only *Bosea* sp. WAO, and *Bosea* sp. 117 genomes contain both the large and small arsenite subunits with an amino acid similarity of 78% for AioA and 73% for AioB. The genes within the arsenite oxidation operon are in the same order (Fig. 9). The operon begins with a sensor histidine kinase, *aioS*, followed by a transcriptional response regulator, *aioR*, and then *aioB*, followed by *aioA*.

#### Reduced sulfur compound oxidation

**Fig. 10:**
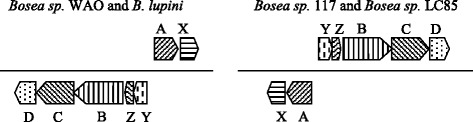
Operon structure for the *sox* genes for thiosulfate oxidation. The orientation is 5′-3′ with the plus strand on top. The orientation of the genes for *Bosea* sp. WAO and *Bosea lupini* are the same while *Bosea* sp. LC85 and *Bosea* sp. 117 have the same orientation. These operons are inverted between the plus and minus strands

*Bosea* sp. WAO is also able to grow under chemolithoautotrophic conditions with thiosulfate, polysulfide, and elemental sulfur. Metabolic studies indicated that the organism is able to stoichiometically oxidize the electron donor S_2_O_3_ to SO_4_^2−^. The *sox* gene cluster is a pathway consisting of seven essential genes, *soxXYZABCD,* that code for proteins required for direct oxidation from sulfide to sulfate in vivo [17]. The genome analysis indicated that strain WAO possesses all the genes necessary for the sulfur oxidation pathway. KEGG analysis indicated genes are all present to code for the enzymes SoxB, SoxX, SoxY, SoxA, SoxC, and SoxD to allow for complete oxidation of S_2_O_3_ to SO_4_^2−^. *Bosea* sp. WAO, in addition to *B. thiooxidans* CGMCC 9174 V5_1, *Bosea* sp. 117, *Bosea* sp. LC85, and *B. lupini* contain the complete *sox* system. For the four genomes available in IMG the overall gene order in the operons are the same for all organisms; however, *Bosea* sp. WAO and *B. lupini* have *soxA* and *soxX* on the plus strand and *soxY*, *soxZ*, *soxB*, *soxC*, *soxD* on the minus strand (Fig. 10). While *Bosea* sp. 117 and *Bosea* sp. LC85 have the genes on the reverse strands with *soxY*, *soxZ*, *soxB*, *soxC*, *soxD* on the plus and *soxA* and *soxX* on the minus strand (Fig. 10). Comparison of the translated nucleotide sequence of *soxB* from *Bosea* sp. WAO to the translated *soxB* of the other five organisms showed that the protein sequence is 90% similar to *Bosea* sp. LC85, 88% similar to *B. lupini* and *B. thiooxidans* CGMCC 9174 V5_1, and 70% similar to *Bosea* sp. 117. The presence of all the genes in the same order suggests other strains in addition to the experimentally confirmed *Bosea thiooxidans* BI-42^T^, may be able to perform thiosulfate oxidation.

#### Additional metabolic pathways

The Calvin Cycle consists of 13 enzymatic reactions with the enzyme ribulose-1,5 bisphosphate carboxylase/oxygenase (RuBisCO) responsible for the carbon fixation step [18]. For the initial publication of *Bosea* sp. WAO the type II ribulose-1,5’bisphosphate carboxylase/oxygenase (RuBisCO) was amplified by traditional PCR [1, 16]. Analysis for the remaining genes of the Calvin-Benson-Bassham Cycle for carbon fixation indicated that all the other required genes were present for carbon fixation to occur. Nine of the available genomes have a match for strain WAO’s ribulose 1,5-bisphosphate carboxylase amino acid sequence: *B. thiooxidans* CGMCC 9174 V5_1, (85%), *B. lathyri* DSM 26656^T^, (86%), *B. lupini* DSM 26673^T^, (82%), *B. vaviloviae* strain SD260, (85%), *Bosea* sp. 117, (72%), *Bosea* sp. UNC402CLCol, (85%), *Bosea* sp. LC85, (84%), *Bosea* sp. OK403, (87%), and *Bosea* *sp*. AAP35, (84%). Since RuBisCO is considered a biomarker for the Calvin Cycle this suggests carbon fixation maybe be widespread in this genus despite the limited experimental evidences.

Additional KEGG analysis indicated incomplete pathways for nitrogen reduction. *Bosea* sp. WAO possesses some genes for each of the reductive pathways but each is incomplete supporting the observation that no growth occurred when nitrate was provided as an electron acceptor. No genes involved in ammonia oxidation were identified again supporting the absence of growth when cultivated under those conditions [1]. Using IMG/ER Pipeline analysis *Bosea* sp. WAO was determined to be prototrophic for L-aspartate, L-glutamate, and glycine; auxotrophic for L-lysine, L-alanine, L-phenylalanine, L-tyrosine, L-tryptophan, L-histine, L-arginine, L-isoleucine, L-leucine, and L-valine; and not able to synthesize selenocycteine synthesizer or biotin based on the draft of the genome [9]. Using the SEED viewer *Bosea* sp. WAO has complete pathways for the: tricarboxylic acid cycle, pentose phosphate pathway, acetyl-coA acetogenesis pathway, methylglyoxal metabolism, dihydroxyacetone kinases, catechol branch of beta-ketoadipate pathway, glycerol and clycerol-3-phosphate uptake and utilization, D-ribose utilization, deoxyribose and deoxynucleoside catabolism, and lactate utilization.

## Conclusions

*Bosea* sp. WAO is able to grow chemolithoautotrophically on both arsenite and reduced sulfur compounds. It was originally enriched from pyritic shale obtained from a rock outcropping containing arsenic in the Lockatong geological formation in the Newark Basin near Trenton, New Jersey [1]. The draft genome is 6.1 Mbps and a G + C content of 66.84%. COG analysis for *Bosea* sp. WAO assigned a large number of genes to amino acid transport and metabolism (13.76%), transcription (8.13%), inorganic ion transport and metabolism (8.06%), and energy production and conservation (6.97%). *Bosea* sp. WAO has 53 genes encoding for cytochromes alone. Strain WAO is able to engage in the oxidative part of biogeochemical cycling and grow autotrophically when nutrient conditions are low. When conditions favor heterotrophic growth, however, the organism is able to rapidly increase in biomass and maintain its population under the varying conditions that expected to prevail at an oxic mineral surface.
